# Molecular Advances in SARS-CoV-2: A Brief Update on Transmission, Infection, and Pathology Aspects

**DOI:** 10.3390/ijms232214250

**Published:** 2022-11-17

**Authors:** João R. Mesquita

**Affiliations:** 1 Instituto de Ciências Biomédicas Abel Salazar (ICBAS), Universidade do Porto, Rua de Jorge Viterbo Ferreira, 228, 4050-313 Porto, Portugal; jrmesquita@icbas.up.pt; Tel.: +351-220-428-000; 2 Epidemiology Research Unit (EPIUnit), Instituto de Saúde Pública da Universidade do Porto (ISPUP), 4050-313 Porto, Portugal

## 1. Introduction

It all started back in late 2019 with a virus making a leap, crossing the barrier of species from an animal reservoir to a human and quickly spreading around the world. The virus went on to infect 625,000,000 individuals and cause over 6,500,000 deaths [1]. This was the most important zoonotic event of the past few years, emerging from Wuhan, China, where this new coronavirus was detected for the first time in patients with acute pneumonia and termed SARS-CoV-2, causing the Coronavirus Disease 2019 or COVID-19 [2]. However, this was not the first occurrence of a zoonosis. Although substantial developments in medical/environmental surveillance and in diagnostic power have been achieved over time, zoonotic emerging and re-emerging diseases remain a major global concern, to such an extent that more than 61% of all human pathogens are zoonotic, representing 75% of all emerging pathogens in the past decade [3].

The pandemic properties of SARS-CoV-2 are, in part, due to the virus transmission, which is known to occur not only in an airborne manner, but also through indirect contact transmission involving the contact of a susceptible host with a contaminated object or surface (fomite transmission) [4]. Over two years have elapsed since the discovery of SARS-CoV-2, and still, information is scarce on the molecular aspects of viral transmission. Hence, a molecular approach to SARS-CoV-2 transmission is of the utmost importance.

This Special Issue of the *International Journal of Molecular Sciences*, “Molecular Advances in SARS-CoV-2 Transmission, Infection and Pathology”, with a total of seven manuscripts, focuses on an important group of aspects related to SARS-CoV-2 and COVID-19, including the most recent advances in the molecular detection and pathology of the disease. Contributions from recognized colleagues have been collated, including original research articles and comprehensive reviews covering the molecular aspects of SARS-CoV-2 transmission, infection, and pathology.

## 2. Reviews

The Issue opens with two interesting reviews, the first by Ramasamy [5], which highlights the most recent body of data on the value of proper humidification (conditioning) and warming of inhaled air, in order to reduce the viral infectivity of SARS-CoV-2 in the upper respiratory tract. The paper first addresses the relevance of SARS-CoV-2 airborne transmission, followed by a reflection on the nasal epithelium as the initial SARS-CoV-2 replication site, and the impact of type 1 and 3 interferons on preventing viral infection in the upper respiratory tract epithelial cells. The manuscript then addresses the pertinent aspects of weaker innate immune responses to respiratory viral infections in upper respiratory tract epithelial cells at sub-optimal temperature and humidity, and the early innate immune responses in the upper respiratory tract for limiting and eliminating SARS-CoV-2 infections. Ramasamy concludes that the currently available data support that severe COVID-19 can be mitigated with optimal nasal air conditioning, in turn, reducing SARS-CoV-2 infectivity of the upper respiratory tract and, as a consequence, severe COVID-19. Further studies on SARS-CoV-2 infection rates and viral loads in the nasal cavity and nasopharynx in relation to inhaled air temperature, humidity, age, gender, and genetic background are needed in this context. This review concludes that face masks used for reducing airborne virus transmission can also boost improved nasal air conditioning in cold weather, thereby also minimizing SARS-CoV-2 infectivity.

The second review of this Special Issue is authored by Queirós-Reis et al. [6] and emphasizes the pertinence of SARS-CoV-2 cell infection mediated by a densely glycosylated spike protein, a fusion protein, binding human angiotensin-converting enzyme 2 (hACE2), which acts as the functional receptor through the receptor-binding domain (RBD). The authors explore the interaction of hACE2 with the RBD, exploring how fusion is commenced. Additionally, infectivity and immune response mediation by occurring mutations is also addressed. For this, the authors focus on all structures available at that time in the Protein Data Bank for the interaction between SARS-CoV-2 S protein and hACE2 that show the particular relevance of the Delta variant, with particular interest in mutations that were associated with increased viral fitness through decreased antibody binding, increased RBD affinity, and altered protein dynamics. The impact and value of merging data on both existing mutations and mutagenesis studies are underlined by the predictive power of novel potential SARS-CoV-2 variants that harbor advantageous spike protein mutations, including mutations S13I and W152C, decreasing antibody binding; N460K, increasing RDB affinity; or Q498R, positively affecting both properties.

## 3. A Broad Variety of Original Research

The Issue concludes with five complete research papers that cover a wide-ranging collection of the molecular aspects of SARS-CoV-2 transmission, infection, and pathology. The first is authored by Gabanella et al. [7] and describes the optimization of a padlock assay to identify SARS-CoV-2 genomic and sub-genomic regions, using formalin-fixed paraffin-embedded placental samples obtained from an individual confirmed to have COVID-19. Using this novel diagnostic approach, SARS-CoV-2 RNA was observed in trophoblastic cells. Additionally, the virion was also observed by immunolocalization of its glycoprotein spike, and the mitochondria of placental villi was imaged, keeping in mind that the mitochondrion has been suggested as a potential residence of the SARS-CoV-2 genome. Overall, the authors observe substantial overlapping of SARS-CoV-2 RNA and mitochondria in trophoblastic cells, correlating with an aberrant mitochondrial network. To the best of the authors’ knowledge, this is the first study showing evidence of the colocalization of SARS-CoV-2 genome and mitochondria in infected tissue.

The second research study, authored by Nagashima et al. [8], initially denotes that mast cells play an important role in inflammatory and vascular hyperpermeability events, demonstrating action on the kallikrein–kinin system, including in patients inflicted by the severe form of COVID-19. Observing a higher number of activated mast cells present in COVID-19 patients and their association with vascular hyperpermeability events, the authors assess factors that lead to the activation and degranulation of these cells and their harmful effects on the alveolar septum environment provided by the action of its mediators. For this, pyroptotic processes throughout caspase-1 (CASP-1) and alarmin interleukin-33 (IL-33) secretion are studied, together with the immunoexpression of ACE2, bradykinin receptor B1 (B1R), and bradykinin receptor B2 (B2R) on post-mortem lung samples from patients affected by COVID-19. The data produced are compared to patients affected by H1N1pdm09 and control patients and show, as a result of the inflammatory processes, the activation of IgE and degranulation of tryptase, as well as Toluidine Blue metachromatic (TB)-stained mast cells of the interstitial and perivascular regions of the same groups. Increased immunoexpression of tissue biomarkers CASP-1, IL-33, ACE2, B1R, and B2R is observed in the alveolar septum of COVID-19 patients, which is associated with a higher density of IgE+ mast cells, tryptase+ mast cells, and TB-stained mast cells, in addition to the presence of intra-alveolar edema. Altogether, the results suggest the direct correlation of mast cells with vascular hyperpermeability, edema, and diffuse alveolar damage events that affect patients with the severe form of disease. The role of kallikrein–kinin system activation in events involving an exacerbated increase in vascular permeability and its direct link with the conditions that precede intra-alveolar edema, as well as the consequent diffuse alveolar damage, is evidenced. The authors ultimately conclude that therapy with activation/degranulation of mast cells-inhibiting drugs can prevent the worsening of the disease and provide a better outcome for the COVID-19 patient.

The third research study, authored by Ferron et al. [9], assessed whether hydroxychloroquine (HCQ)—used in the clinical trial HARMONICOV to treat COVID-19 patients, including obese patients—could have an impact on the metabolism and hepatotoxicity of obese patients using an in vivo/in vitro approach. For this, liquid chromatography high-resolution mass spectrometry in combination with untargeted screening and molecular networking was used to assess drug metabolism in vivo (patient’s plasma) and in vitro (HepaRG cells and RPTEC cells). Moreover, the HepaRG cell model was used to mimic the pathophysiological features of obese patient metabolism, i.e., in the condition of hepatic steatosis. The metabolic signature of HCQ was modified in HepaRG cells cultured under a steatosis condition, and a new metabolite was detected (carboxychloroquine). The RPTEC model was found to produce only one metabolite. A higher cytotoxicity of HCQ was observed in HepaRG cells exposed to exogenous fatty acids, while neutral lipid accumulation (steatosis) was further enhanced in these cells. Finally, in vitro data were compared with the biological parameters of COVID-19 patients treated with HCQ, suggesting that steatosis may be a risk factor for altered drug metabolism and, possibly, toxicity of HCQ.

The fourth study, published by Alzahrani et al. [10], highlights the lack of knowledge on the extracellular vesicles’ (EVs) metabolite content that might play a crucial role during COVID-19, by performing untargeted metabolomics in order to identify the key metabolites and associated pathways that are present in Evs isolated from COVID-19 patients’ sera. The results show that both antivirals and antibiotics—such as Foscarnet, Indinavir, and lymecycline—were found in Evs from individuals treated with these drugs. Additionally, increased levels of anti-inflammatory metabolites, including LysoPS, 7-α,25-Dihydroxycholesterol, and 15-d-PGJ2 were also found in EVs from COVID-19 patients. On the other hand, decreased levels of metabolites associated with coagulation, such as thromboxane and elaidic acid, were found in EVs from COVID-19 patients. Altogether, the data from this study suggest that EVs not only carry active drug molecules, but also anti-inflammatory metabolites, suggesting that exosomes might play a role in negotiating with heightened inflammation during COVID-19.

The last study, authored by Cai et al. [11], screens the gene expression of three host receptors (ACE2, DC-SIGN, and L-SIGN) of SARS coronaviruses and dendritic cells (DCs) status in bulk and single-cell transcriptomic datasets of the upper airway, lung, or blood of COVID-19 patients and healthy controls. In COVID-19 patients, DC-SIGN gene expression was found to be decreased in lung DCs but increased in blood DCs. Conventional DCs were depleted, while plasmacytoid DCs were augmented in the lungs of mild COVID-19. In severe cases, augmented types of immature DCs (CD22+ or ANXA1+ DCs) with MHCII downregulation were observed. Overall, the data from this study indicate that DCs in severe cases stimulate innate immune responses but fail to specifically present SARS-CoV-2.

## 4. Concluding Remarks

This is a brief but assertive collection that showcases the need to address the molecular aspects of SARS-CoV-2 transmission, infection, and pathology. The global perspective, highlighted by the contents here presented, strengthens the need to address joint and wide efforts in these topics.

## References

[1] (2022). WHO. https://covid19.who.int/.

[2] Morens D.M., Fauci A.S. (2020). Emerging Pandemic Diseases: How We Got to COVID-19. Cell.

[3] Jones K.E., Patel N.G., Levy M.A., Storeygard A., Balk D., Gittleman J.L., Daszak P. (2008). Global trends in emerging infectious diseases. Nature.

[4] Da Silva P.G., Nascimento M.S.J., Soares R.R., Sousa S.I., Mesquita J.R. (2020). Airborne spread of infectious SARS-CoV-2: Moving forward using lessons from SARS-CoV and MERS-CoV. Sci. Total Environ..

[5] Ramasamy R. (2021). Perspective of the Relationship between the Susceptibility to Initial SARS-CoV-2 Infectivity and Optimal Nasal Conditioning of Inhaled Air. Int. J. Mol. Sci..

[6] Queirós-Reis L., da Silva P.G., Gonçalves J., Brancale A., Bassetto M., Mesquita J.R. (2021). SARS-CoV-2 Virus−Host Interaction: Currently Available Structures and Implications of Variant Emergence on Infectivity and Immune Response. Int. J. Mol. Sci..

[7] Gabanella F., Barbato C., Corbi N., Fiore M., Petrella C., de Vincentiis M., Greco A., Ferraguti G., Corsi A., Ralli M. (2022). Exploring Mitochondrial Localization of SARS-CoV-2 RNA by Padlock Assay: A Pilot Study in Human Placenta. Int. J. Mol. Sci..

[8] Nagashima S., Dutra A.A., Arantes M.P., Zeni R.C., Klein C.K., de Oliveira F.C., Piper G.W., Brenny I.D., Pereira M.R.C., Stocco R.B. (2022). COVID-19 and Lung Mast Cells: The Kallikrein–Kinin Activation Pathway. Int. J. Mol. Sci..

[9] Ferron P.-J., Le Daré B., Bronsard J., Steichen C., Babina E., Pelletier R., Hauet T., Morel I., Tarte K., Reizine F. (2021). Molecular Networking for Drug Toxicities Studies: The Case of Hydroxychloroquine in COVID-19 Patients. Int. J. Mol. Sci..

[10] Alzahrani F.A., Mohammed M.R.S., Alkarim S., Azhar E.I., El-Magd M.A., Hawsawi Y., Abdulaal W.H., Yusuf A., Alhatmi A., Albiheyri R. (2021). Untargeted Metabolic Profiling of Extracellular Vesicles of SARS-CoV-2-Infected Patients Shows Presence of Potent Anti-Inflammatory Metabolites. Int. J. Mol. Sci..

[11] Cai G., Du M., Bossé Y., Albrecht H., Qin F., Luo X., Androulakis X.M., Cheng C., Nagarkatti M., Nagarkatti P. (2021). SARS-CoV-2 Impairs Dendritic Cells and Regulates DC-SIGN Gene Expression in Tissues. Int. J. Mol. Sci..

